# Further insights into the eco-epidemiology of American cutaneous leishmaniasis in the Belem metropolitan region, Pará State, Brazil

**DOI:** 10.1590/0037-8682-0255-2020

**Published:** 2020-12-11

**Authors:** Lucas Pantoja Gonçalves, Thiago Vasconcelos dos Santos, Marliane Batista Campos, Luciana Vieira do Rêgo Lima, Edna Aoba Yassui Ishikawa, Fernando Tobias Silveira, Patrícia Karla Santos Ramos

**Affiliations:** 1 Instituto Evandro Chagas, Laboratório de Leishmanioses ‘Prof. Dr. Ralph Lainson’, Seção de Parasitologia, Ananindeua, PA, Brasil.; 2 Universidade Federal do Pará, Núcleo de Medicina Tropical, Belém, PA, Brasil.

**Keywords:** American cutaneous leishmaniasis, *Leishmania* spp, Molecular characterization, PCR-RFLP, Amazonian Brazil

## Abstract

**INTRODUCTION:**

In the Belém Metropolitan Region (BMR), Pará State, Brazil, American cutaneous leishmaniasis (ACL) is endemic; however, very little is known regarding its causative agents. Therefore, we used our standard diagnostic approach combined with an RNA polymerase II largest subunit (RNAPOIILS)-polymerase chain reaction (PCR) followed by analysis of restriction fragment length polymorphism (PCR-RFLP) to identify *Leishmania* spp. ACL agents in this region.

**METHODS:**

Thirty-two *Leishmania* spp. isolates from patients with ACL in the BMR during 1995-2018 were analyzed. *Leishmania* spp. DNA samples were amplified using the primers RPOR2/RPOF2, and the 615-bp PCR products were subjected to enzymatic digestion using *TspRI* and *HgaI* endonucleases.

**RESULTS:**

ACL etiological agents in the BMR comprised *Leishmania (Viannia) lindenbergi* (43.7%) followed by *Leishmania (Viannia) lainsoni* (34.4%), *Leishmania (Leishmania) amazonensis* (12.5%), and *Leishmania (Viannia) braziliensis* (9.4%).

**CONCLUSIONS:**

To our knowledge, the results of the study revealed for the first time that *L. (V.) lindenbergi* and *L. (V.) lainsoni* are the main ACL agents in BMR.

## INTRODUCTION

American cutaneous leishmaniasis (ACL) is a parasitic protozoan disease caused by different Leishmaniinae parasites (Kinetoplastida: Trypanosomatidae) and is widely distributed in most Latin American countries. There are at least 15 recognized species within the subgenera *Leishmania* (*Leishmania*), *L.* (*Viannia*)*,* and *L.* (*Mundinia*) that may give rise to human diseases^1^ ^,^ ^2^. Seven well-known *Leishmania* species have been identified as ACL agents in Brazil; *Leishmania (V.) braziliensis*, *L.* (*V.*) *guyanensis*, *L. (V.) lainsoni*, *L.* (*V.*) *shawi*, *L.* (*V.*) *naiffi*, *L.* (*V.*) *lindenbergi,* and *L.* (*L.*) *amazonensis* ^3^. More recently, a new subspecies, *L.* (*V.*) *shawi santarensis*, as well as the first hybrid parasite, *L.* (*V.*) *guyanensis* **/** *L.* (*V.*) *shawi shawi*, have been found in the Brazilian Amazon^4^.

ACL behaves as a primary zoonosis of wild mammals in Brazil, and the transmission of *Leishmania* species occurs through the bites of infected females of different phlebotomine vectors (Diptera: Psychodidae)^5^ ^-^ ^8^. ACL has an occasional but endemic character in the Belém Metropolitan Region (BMR), Pará State, in the Brazilian Amazon that is mainly associated with three *Leishmania* species, including *L.* (*L.*) *amazonensis* ^9^, *L.* (*V.*) *lainsoni* ^10^, and *L.* (*V.*) *lindenbergi* ^11^. Over the years, the BMR has experienced an increase in growth rate with intense urban construction and displacement of populations to areas neighboring secondary native forest areas, favoring human contact with the enzootic cycles of these *Leishmania* species.

The identification of potential ACL agents is a key step in surveillance strategies, and the existing knowledge and molecular tools available for the identification and characterization of *Leishmania* species must be improved and harmonized^12^. In this sense, species typing has evolved into a molecular approach. An overview of the different methods and targets currently available can be found elsewhere^13^.

Polymerase chain reaction (PCR) followed by restriction fragment length polymorphism (RFLP) has been widely applied to characterize New World *Leishmania* species, and has been focused on different targets of kinetoplast or genomic DNA^14^ ^-^ ^19^. Due to the high inter/intraspecific diversity/polymorphism in the parasites, these techniques do not usually show continental reproducibility, and regional-scale assays must be improved to validate established protocols. To this end, a set of targets that encode the genes of the RNA polymerase II largest subunit (RNAPOIILS; considered phylogenetically informative as defined by parsimony criteria) has been used to explore the relationships between *Leishmania* species^20^. We used our standard diagnostic approach combined with the RNAPOIILS-PCR-RFLP assay (previously designed to identify Amazonian/Guianan *Leishmania* species)^19^ to identify *Leishmania* spp. that act as potential ACL agents in the BMR. These results provide crucial new insights into the eco-epidemiology of ACL in this region.

## METHODS

### Study area

**FIGURE 1:**
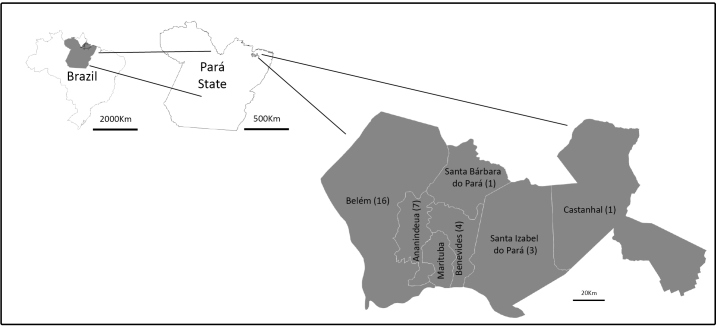
Study area; the Belém Metropolitan Region, located in the northeastern Pará State, Brazil. ( ) number of *Leishmania* isolates (1995-2018) in each municipality.

The BMR comprises a cluster of socioeconomic integrated municipalities located in the northeastern Pará State, Brazil (Belém [the State capital], Ananindeua, Marituba, Benevides, Santa Izabel do Pará, Santa Bárbara do Pará, and Castanhal), with a resident population of approximately 2,505,242 inhabitants^21^ and a territorial area of 6.890 km² (Figure 1). The landscape has an extensive alluvial plain, with a typically equatorial climate and average temperatures ranging from 24°C to 26°C and humidity above 80%. The annual precipitation is approximately 2500 mm, with a rainy season from January to June. The vegetation is mainly secondary forest, although some original remnants still cover ~31% of the region, which is composed of upland (*terra firme*), floodplain (*várzea*), and wetland (*igapó*) forests^22^.

### Surveying the ACL epidemiology in the BMR

Patients with ACL examined at the Ralph Lainson Leishmaniases Lab (with the BMR as the geographical local of presumed infection from 1995 to 2018) were also screened following our standard diagnostic approach comprising clinical-epidemiological investigation and laboratory diagnosis. The patients were diagnosed by parasitological demonstrations (Giemsa-stained smears of exudates from cutaneous lesions) and by the interpretation of the Montenegro skin test (inactivated promastigotes of *L.* (*V.*) *braziliensis* - MHOM/BR/M17323 - 1×10^7^ parasites/mL), as previously described^4^ ^,^ ^23^ ^,^ ^24^. *In vitro/in vivo* parasite isolation (inoculating exudates from cutaneous lesions in Difco B^45^ media and/or in the hind foot of *Mesocricetus auratus*) was also performed routinely^25^. The ACL-confirmed cases received systemic therapy (meglumine antimoniate) at a dose of 12 mg Sb5/kg/day in two series of 22 days, with an interval of 7-10 days between each series^23^.

All *Leishmania* spp. isolates obtained from ACL cases of localized cutaneous leishmaniasis (LCL) clinical form originating from the BMR from 1995 to 2018 were included in the analysis. Of the 32 cultured samples, 16 were from the municipality of Belém, seven from Ananindeua, four from Benevides, three from Santa Izabel, one from Santa Bárbara do Pará, and one from Castanhal. No isolate was registered in the municipality of Marituba (Figure 1).

### DNA extraction/quantification

DNA was extracted from positive culture samples using the commercial Reliaprep gDNA Tissue Miniprep System Kit (Promega, USA). After performing cell lysis using proteinase K, the samples were placed in a column surrounded by a silica membrane, and during centrifugation, the DNA adhered to the membrane. After several washes, the extracted DNA was eluted in 150 μL of elution buffer. DNA sample quantification was performed using a Qubit 2.0 fluorometer (Invitrogen, USA).

### RNAPOIILS-PCR-RFLP

The methodology was adapted from Simon et al.^19^ with minor modifications. In brief, *Leishmania* DNA amplification was performed using the primers RPOF2 (5′-AGAACATGGGCGGCC-3′) and RPOR2 (5′-CGAGGGTCACGTTCTTG-3′), which amplified a 615-bp fragment of the RNAPOIILS gene. The reaction was performed in a final volume of 50 μL containing 0.2 μM of each DNTP (dATP, dCTP, dGTP, and dTTP) (Quatro G), 0.01 μM of each primer (Invitrogen), 2.5 U of Taq DNA polymerase (Invitrogen), and 10 μL of extracted DNA (1 ng/μL). The reactions were performed in an Eppendorf (Mastercycler® personal) thermal cycler programmed for an initial denaturation temperature of 94°C for 5 min, followed by 40 cycles of 94°C for 30 s, 55°C for 30 s, and 72°C for 1 min. The final extension step was maintained for 5 min at 72°C. The PCR products were applied to a 1% agarose gel and stained with Safe Dye (Kasvi) to confirm proper amplification. 

A total of 15 μL of the PCR product was digested with 10 U of *Tsp*RI (New England Biolabs) (2 h at 65°C) or with 2 U of *Hga*I (New England Biolabs) endonucleases (2 h at 37°C), following the manufacturer's recommendations. Both digestions (*Hga*I and *Tsp*RI) were performed separately for each 15 μL of PCR product in a final volume of 20 μL. The digestion mixtures were individually applied to 3% agarose gels and stained with Safe dye. 

### Molecular characterization of *Leishmania* spp. isolates

The molecular characterization of *Leishmania* spp. isolates from patients with ACL in the BMR was based on the RNAPOIILS-PCR-RFLP analysis in accordance with previously published studies^19^ ^,^ ^30^. The following World Health Organization (WHO) *Leishmania* reference strains preserved in the Ralph Lainson Leishmaniases Lab (Instituto Evandro Chagas) cryobank that had previously been characterized were selected for this analysis: *L.* (*L.*) *amazonensis* (IFLA/BR/1967/PH8), *L.* (*V.*) *braziliensis* (MHOM/BR/1975/M2904), *L.* (*V*.) *guyanensis* (MHOM/BR/1975/M4147), *L.* (*V.*) *naiffi* (MDAS/BR/1979/M5533), *L.* (*V.*) *lainsoni* (MHOM/BR/1981/M6426), *L.* (*V.*) *shawi* (MCEB/BR/1984/M8408), and *L.* (*V*.) *lindenbergi* (MHOM/BR/1998/M15732)^4^. However, considering the known genetic diversity of some *Leishmania* species in the Brazilian Amazon, especially *L.* (*V*.) *braziliensis* and *L.* (*V.*) *guyanensis*, another strain of *L.* (*V.*) *braziliensis* (MHOM/BR/1975/M2903), which differs from the above reference strains, was included in this analysis as well as another strain of *L.* (*V*.) *guyanensis* (MHOM/BR/1997/M16342)(Table 1).

**TABLE 1:** *Leishmania* spp. WHO reference strains (and closely related others) used for the RNA polymerase II largest subunit (RNAPOIILS) - polymerase chain reaction followed by analysis of restriction fragment length polymorphism (PCR-RFLP) and their respective digestion profiles with TspRI and HgaI endonucleases

Species	Host	WHO code	Locality	RNAPOIILS-PCR-RFLP profile
*L.* (*V.*) *braziliensis*	*Homo sapiens*	MHOM/BR/1975/M2903	Parauapebas - PA	TspRI SG3 - HgaI SG6
*L.* (*V.*) *braziliensis*	*Homo sapiens*	MHOM/BR/1975/M2904	Parauapebas - PA	TspRI SG3 - HgaI SG2*
*L.* (*V.*) *guyanensis*	*Homo sapiens*	MHOM/BR/1975/M4147	Almeirim - PA	TspRI SG2 - HgaI SG2
*L.* (*V.*) *guyanensis*	*Homo sapiens*	MHOM/BR/1997/M16342	Rio Preto da Eva - AM	TspRI SG2 - HgaI SG2
*L.* (*V.*) *lainsoni*	*Homo sapiens*	MHOM/BR/1981/M6426	Benevides - PA	TspRI SG3 - HgaI SG4
*L.* (*V.*) *naiffi*	*Dasypus novemcintus*	MDAS/BR/1979/M5533	Almeirim - PA	TspRI SG3 - HgaI SG3
*L.* (*V.*) *shawi*	*Sapajus apella*	MCEB/BR/1984/M8408	Parauapebas - PA	TspRI SG3 - HgaI SG5
*L.* (*V.*) *lindenbergi*	*Homo sapiens*	MHOM/BR/1996/M15729	Belém - PA	TspRI SG3 - HgaI SG2*
*L.* (*L.*) *amazonensis*	*Bichromomyia flaviscutellata*	IFLA/BR/1967/PH8	Belém - PA	TspRI SG1- HgaI SG1

*L*.: *Leishmania*; *V*.: *Viannia*; **WHO:** World Health Organization; **PA:** Pará State, Brazil; **AM:** Amazonas State, Brazil. *These different *Leishmania* species present the same RNAPOIILS-PCR-RFLP profile, requiring additional methods for unambiguous characterization.

**FIGURE 2:**
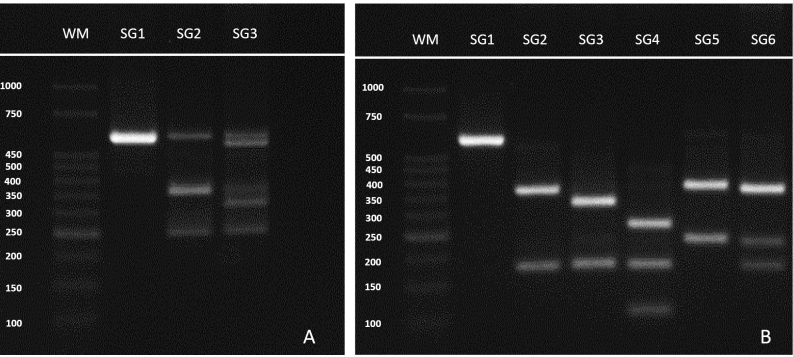
RNA polymerase II largest subunit (RNAPOIILS)-polymerase chain reaction followed by analysis of restriction fragment length polymorphism (PCR-RFLP) of *Leishmania* spp. digestion profiles (TspRI and HgaI endonucleases). **(A):** TspRI digestion subgroups (SGs): WM, molecular weight marker (in base pairs) (1kb-Ludwig Biotec); SG1 (no restriction site), corresponding to *L.* (*L.*) *amazonensis;* SG2, consisting of *L.* (*V*.) *guyanensis*; and SG3, consisting of *L.* (*V*.) *braziliensis*, *L.* (*V.*) *naiffi*, *L.* (*V.*) *lainsoni*, *L.* (*V*.) *lindenbergi*, and *L.* (*V*.) *shawi*. **(B):** HgaI digestion SGs: SG1 (no restriction site), corresponding to *L.* (*L.*) *amazonensis;* SG2, consisting of *L.* (*V*.) *guyanensis*, *L.* (*V*.) *braziliensis* (MHOM/BR/1975/M2904), and *L.* (*V*.) *lindenbergi*; SG3, consisting of *L.* (*V.*) *naiffi;* SG4, consisting of *L.* (*V.*) *lainsoni*; SG5, consisting of *L.* (*V*.) *braziliensis* (MHOM/BR/1975/M2903); and SG6, consisting of *L.* (*V*.) *shawi*.

Molecular characterization through RNAPOIILS-PCR-RFLP analysis was based on the reactivity profile of the above WHO *Leishmania* reference strains for both *Tsp*RI and *Hga*I endonucleases. Therefore, *Tsp*RI digestion identified three subgroups (SGs): SG1 (no restriction site), corresponding to *L.* (*L.*) *amazonensis*; SG2, consisting of *L.* (*V*.) *guyanensis*; and SG3, consisting of *L.* (*V*.) *braziliensis*, *L.* (*V.*) *naiffi*, *L.* (*V.*) *lainsoni*, *L.* (*V*.) *lindenbergi*, and *L.* (*V*.) *shawi*, while *Hga*I digestion identified six SGs: SG1 (no restriction site), corresponding to *L.* (*L.*) *amazonensis*; SG2, consisting of *L.* (*V*.) *guyanensis*, *L.* (*V*.) *braziliensis* (MHOM/BR/1975/M2904), and *L.* (*V*.) *lindenbergi*; SG3, consisting of *L.* (*V.*) *naiffi*; SG4, consisting of *L.* (*V.*) *lainsoni*; SG5, consisting of *L.* (*V*.) *shawi*; and SG6, consisting of *L.* (*V*.) *braziliensis* (MHOM/BR/1975/M2903). All seven reference strains could be distinguished from one another using the combined analysis of *Tsp*RI and *Hga*I digestion profiles, with the exception of *L.* (*V*.) *braziliensis* (MHOM/BR/1975/M2904) and *L.* (*V*.) *lindenbergi* (Table 1 and Figure 2).

## RESULTS

### Clinical and epidemiological features of ACL in the BMR

Thirty-two *Leishmania* spp. isolates were obtained within the historical series analyzed from cutaneous lesions of the LCL clinical form of patients with ACL. Most lesions (75%; 24/32) were single (ranging from to 1-2) and localized to the arm and/or leg (81%; 26/32), with reactive Montenegro skin tests ranging at 5-32 mm. The ACL sample comprised patients with a mean age of 32.5 ±18.9 years, predominantly male (81%; 26/32), with histories of activities in forested areas (Table 2). All ACL-confirmed cases showed satisfactory treatment responses with no history of relapse for one year post-treatment.

**TABLE 2:** *Leishmania* spp. isolates from patients with American cutaneous leishmaniasis from the Belém Metropolitan Region (1995-2018), characterized by RNA polymerase II largest subunit (RNAPOIILS)-polymerase chain reaction followed by analysis of restriction fragment length polymorphism (PCR-RFLP).

Mnemonic	Infection site	Sex	Age	Lesions	MST (mm)	WHO code	RNAPOIILS-PCR-RFLP profile	***Leishmania*species**
SNFS	Ananindeua	M	22	2 (leg)	n.a.	MHOM/BR/1995/M15265	TspRI SG3 - HgaI SG4	*L*. (*V*.) *lainsoni*
EBR	Ananindeua	F	17	1 (leg)	n.a.	MHOM/BR/1995/M15418	TspRI SG1- HgaI SG1	*L*. (*V*.) *amazonensis*
LSM	Ananindeua	M	21	1 (leg)	n.a.	MHOM/BR/1996/M15720	TspRI SG1- HgaI SG1	*L*. (*V*.) *amazonensis*
JMS	Belém	M	20	1 (arm)	17 x 17	MHOM/BR/1996/M15279	TspRI SG3 - HgaI SG2	*L*. (*V*.) *lindenbergi*
OPR	Benevides	M	19	2 (hand)	32 x 32	MHOM/BR/1996/M15732	TspRI SG3 - HgaI SG2	*L*. (*V*.) *lindenbergi*
WNSS	Belém	M	19	1 (arm)	n.a.	MHOM/BR/1996/M15740	TspRI SG3 - HgaI SG2	*L*. (*V*.) *lindenbergi*
AOM	Belém	M	20	1 (leg)	10 x 10	MHOM/BR/1996/M15819	TspRI SG3 - HgaI SG2	*L*. (*V*.) *lindenbergi*
PCB	Belém	M	43	1 (back)	n.a.	MHOM/BR/1999/M18048	TspRI SG3 - HgaI SG4	*L*. (*V*.) *lainsoni*
LFB	Belém	F	38	1 (arm)	7 x 9	MHOM/BR/1999/M18054	TspRI SG3 - HgaI SG4	*L*. (*V*.) *lainsoni*
PGP	Belém	M	50	2 (leg)	12 x 12	MHOM/BR/2000/M18820	TspRI SG3 - HgaI SG2	*L*. (*V*.) *lindenbergi*
MDSA	Belém	M	20	1 (arm)	n.a.	MHOM/BR/2000/M19418	TspRI SG3 - HgaI SG2	*L*. (*V*.) *lindenbergi*
WGH	Benevides	M	41	2 (leg)	8 x 8	MHOM/BR/2000/M19477	TspRI SG3 - HgaI SG6	*L*. (*V*.) *braziliensis*
VLS	Castanhal	M	21	1 (arm)	n.a.	MHOM/BR/2000/M19480	TspRI SG3 - HgaI SG6	*L*. (*V*.) *braziliensis*
PAN	Santa Barbara	M	45	1 (arm)	9 x 9	MHOM/BR/2001/M20321	TspRI SG3 - HgaI SG2	*L*. (*V*.) *lindenbergi*
JBS	Santa Izabel	M	32	1 (nose)	n.a.	MHOM/BR/2002/M21484	TspRI SG3 - HgaI SG4	*L*. (*V*.) *lainsoni*
LSB	Ananindeua	M	16	1 (leg)	14 x 14	MHOM/BR/2003/M22090	TspRI SG3 - HgaI SG4	*L*. (*V*.) *lainsoni*
FDS	Belém	M	30	2 (leg)	10 x 10	MHOM/BR/2009/M26488	TspRI SG3 - HgaI SG2	*L*. (*V*.) *lindenbergi*
EAO	Belém	M	44	1 (head)	10 x 10	MHOM/BR/2011/M28690	TspRI SG3 - HgaI SG4	*L*. (*V*.) *lainsoni*
EMO	Belém	M	44	1 (leg)	10 x 10	MHOM/BR/2012/M29080	TspRI SG3 - HgaI SG4	*L*. (*V*.) *lainsoni*
DGA	Benevides	M	20	2 (arm)	10 x 10	MHOM/BR/2012/M29084	TspRI SG3 - HgaI SG2	*L*. (*V*.) *lindenbergi*
ELG	Belém	M	25	1 (face)	14 x 14	MHOM/BR/2013/M29809	TspRI SG3 - HgaI SG4	*L*. (*V*.) *lainsoni*
SGA	Benevides	F	62	1 (leg)	8 x 8	MHOM/BR/2013/M29986	TspRI SG3 - HgaI SG2	*L*. (*V*.) *lindenbergi*
OMP	Ananindeua	M	33	1 (leg)	13 x 14	MHOM/BR/2013/M30178	TspRI SG3 - HgaI SG2	*L*. (*V*.) *lindenbergi*
CPL	Belém	M	7	2 (face/arm)	5 x 5	MHOM/BR/2014/M30464	TspRI SG3 - HgaI SG4	*L*. (*V*.) *lainsoni*
HMM	Belém	F	19	1(leg)	n.a.	MHOM/BR/2014/M30800	TspRI SG1- HgaI SG1	*L*. (*V*.) *amazonensis*
THS	Belém	F	79	1 (arm)	15 x 15	MHOM/BR/2014/M30921	TspRI SG3 - HgaI SG2	*L*. (*V*.) *lindenbergi*
WNSC	Belém	M	7	2 (face)	30 x 30	MHOM/BR/2015/M31232	TspRI SG3 - HgaI SG4	*L*. (*V*.) *lainsoni*
RFF	Santa Izabel	M	89	1 (face)	n.a.	MHOM/BR/2015/M31234	TspRI SG3 - HgaI SG4	*L*. (*V*.) *lainsoni*
OSP	Santa Izabel	M	44	1 (arm)	5 x 5	MHOM/BR/2015/M31462	TspRI SG1- HgaI SG1	*L*. (*V*.) *amazonensis*
PLBS	Ananindeua	F	16	1 (leg)	9 x 9	MHOM/BR/2015/M31702	TspRI SG3 - HgaI SG2	*L*. (*V*.) *lindenbergi*
FEGA	Belém	M	41	1 (arm)	12 x 12	MHOM/BR/2016/M31799	TspRI SG3 - HgaI SG6	*L*. (*V*.) *braziliensis*
WFS	Ananindeua	M	36	1 (leg)	10 x 10	MHOM/BR/2018/M32884	TspRI SG3 - HgaI SG2	*L*. (*V*.) *lindenbergi*

**WHO:** World Health Organization; **MST:** Montenegro skin test; **n.a.:** not available.

### Identification of *Leishmania* spp. isolates through RNAPOIILS-PCR-RFLP analysis

**FIGURE 3:**
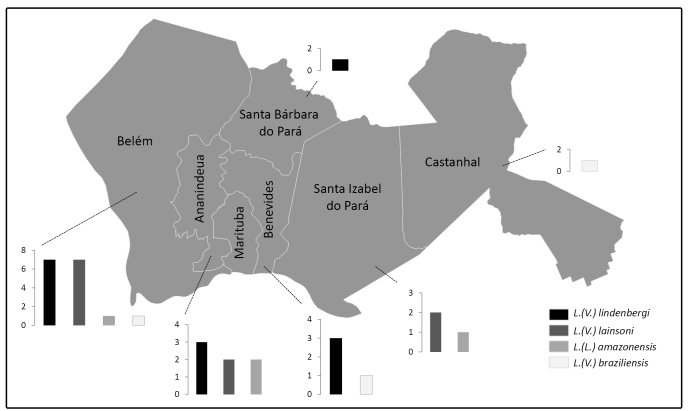
Distribution of *Leishmania* species in the municipalities of the Belém Metropolitan Region.

The RNAPOIILS-PCR-RFLP analysis of *Leishmania* spp. DNA products allowed the identification of 14 (43.7%) isolates of *L. (V.) lindenbergi*, 11 (34.4%) of *L. (V.) lainsoni*, four (12.5%) of *L. (L.) amazonensis*, and three (9.4%) of *L. (V.) braziliensis* (**Table 2**). The geographic distributions of the known presumptive contamination sites showed *Leishmania*species distributions (at the municipality level) as follows: Belém 16 (50%) [*L.*(*V.*)*lindenbergi* seven, *L.* (*V.*) *lainsoni* seven,*L. (L.) amazonensis*one, and *L.* (*V.*) *braziliensis* one], Ananindeua seven (21.8%) [*L.*(*V.*)*lindenbergi* three, *L.* (*V*.) *lainsoni* two, and *L.* (*L.*) *amazonensis* two], Benevides four (12.5%) [*L.*(*V.*)*lindenbergi* three and *L.* (*V.*) *braziliensis* one], Santa Izabel do Pará three (9.5%) [*L.*(*V.*)*lainsoni* two and *L.* (*L*.) *amazonensis* one], Santa Bárbara do Pará one (3.1%) [*L.*(*V.*)*lindenbergi*], and Castanhal one (3.1%) [*L.*(*V.*)*braziliensis*] (Figure 3).

The combined analysis of the *Tsp*RI and *HgaI* digestion profiles of the isolates of *L.* (*V*.) *lindenbergi*, *L.* (*V.*) *lainsoni,* and *L.* (*L.*) *amazonensis* presented the same RNAPOIILS-PCR-RFLP profiles as their respective WHO *Leishmania* reference strains*.* However, considering that the two *L.* (*V*.) *braziliensis* WHO *Leishmania* reference strains (MHOM/BR/1975/M2904 and MHOM/BR/1975/M2903) showed two distinct patterns upon *Hga*I digestion, all *L.* (*V*.) *lindenbergi* isolates could be distinguished from *L.* (*V*.) *braziliensis* MHOM/BR/1975/M2903, but not from the MHOM/BR/1975/M2904 strain. Likewise, all three isolates of *L.* (*V*.) *braziliensis* were identified based on the MHOM/BR/1975/M2903 RNAPOIILS-PCR-RFLP profile (Table 2). To clarify this ambiguous reactivity (*Tsp*RI and *Hga*I) between *L. (V.) lindenbergi* and *L.* (*V*.) *braziliensis* (MHOM/BR/1975/M2904), the biological behavior of experimentally infected hamsters was checked for all 14 isolates of *L.* (*V*.) *lindenbergi* after the parasite was isolated, which revealed that none of these isolates could produce apparent ulcerated cutaneous lesion at the “hamster” inoculation site (hindfoot), suggesting a typical behavior of *L.* (*V*.) *lindenbergi* infection and not of *L.* (*V*.) *braziliensis* infection.

## DISCUSSION

DNA-based methods have been extensively used since the 1980s for the characterization of *Leishmania* spp. However, these methods are currently restricted to referral hospitals and research centers with well-equipped laboratories. Currently available techniques include direct sequencing of a PCR product, use of species-specific restriction sites via PCR-RFLP, PCR fingerprinting, random amplified polymorphic DNA, or high-resolution melting. Of these, only PCR-RFLP and sequence analysis coupled with the appropriate target in the genome are suitable for the discrimination of all *Leishmania* species. In many cases, a combination of different markers must be applied to achieve a definitive taxonomic resolution^13^ ^,^ ^14^.

The characterization of *Leishmania* spp. has traditionally been performed in the Ralph Lainson Leishmaniases Lab (since the 1970s) using a combined methodology that takes into consideration the parasite's behavior within experimentally infected invertebrate (phebotomines) and vertebrate (hamsters) hosts in the Dfico B^45^culture medium, but has been improved with the “gold standard” MLEE and IFAT-Mabs analyses^8^ ^,^ ^26^ ^-^ ^29^. We extended the applicability of a simple and direct molecular tool that was originally proposed (and recently revised) for French Guiana^19^ ^,^ ^30^, and used it to identify (to date) the coexisting human-pathogenic dermotropic *Leishmania* species in the BMR. This methodology has already been used to identify *Leishmania* isolates from patients with ACL in our immunopathology research laboratory^31^ as well as from phlebotomines^24^ ^,^ ^29^. The sensitivity of RNAPOIILS-PCR-RFLP was 100%, as expected for isolates. High specificity was also presumed, since the RNAPOIILS-PCR-RFLP for *Leishmania* profiles is distinct from that for other microorganisms. For non-isolated samples, future steps will include the analysis of Giemsa-stained lesion imprint slides, which have a presumed sensitivity of approximately 90%^19^, to improve sensitivity for *Leishmania* DNA detection. These samples can be preliminarily screened with markers targeting kDNA.

The RNAPOIILS-PCR-RFLP profiles of *L.* (*L.*) *amazonensis*, *L.* (*V*.) *braziliensis*, *L.* (*V*.) *guyanensis*, *L.* (*V.*) *lainsoni*, and *L.* (*V.*) *naiffi* have been published previously^19^, although, to our knowledge, this is the first analysis of *L.* (*V.*) *shawi* and *L.* (*V.*) *lindenbergi*, thus, providing an extension of the species-specific distinction power of this methodology. The analysis of some *Leishmania* species with high polymorphic genetic potential can be challenging. In this sense, *L.* (*V.*) *guyanensis* from eastern and western Amazonia are antigenically distinct when analyzed by IFAT-Mabs^32^, and a degree of intra-specific heterogeneity has been observed in distinct populations from French Guiana using a small subunit and internal transcribed spacer 1 in the rRNA genes-PCR-RFLP analysis^33^. Despite these observations, no intra-specific variation was observed in the RNAPOIILS-PCR-RFLP analysis.

Additionally, in the present study, *L.* (*V.*) *braziliensis*, a widely distributed and potentially polymorphic species^34^ ^,^ ^35^, revealed different RNAPOIILS-PCR-RFLP profiles of two samples from the same geographical area in the “Serra dos Carajás” southeastern region of the Pará State (MHOM/BR/1975/2904 [Serra Norte N1] and MHOM/BR/1975/M2903 [Serra Norte H7]), with the profile of the former being indistinguishable from that of *L.* (*V.*) *lindenbergi*. This indicates a limitation of PCR-RFLP analysis when referring to an ecological region with a considerable genetic diversity of *Leishmania* parasites. This was also observed in another study in the Brazilian Amazon that failed to distinguish *L.* (*V.*) *lindenbergi* from *L.* (*V*.) *guyanensis* through *hsp*70 PCR-RFLP (with HaeIII digestion). In this scenario, the authors utilized 6-phosphogluconate dehydrogenase, glucose-6-phosphate dehydrogenase MLEE, the analysis of partial sequences of *hsp*70, isocitrate dehydrogenase, and mannose phosphate isomerase genes for final identification^36^. However, in the present study, the biological behavior (experimental infection in “hamster”) of all 14 isolates of suspected *L.* (*V*.) *lindenbergi* when the parasite was isolated revealed that none of these isolates could produce conspicuous ulcerated cutaneous lesions at the inoculation site (footpad) of laboratory animals, thus, confirming the typical biological behavior of *L.* (*V.*) *lindenbergi* infection. Another point that contradicts this high number (14) of isolates being *L*. (*V.*) *braziliensis* is that in over 40 years of research at the BMR, we have never seen a case of mucocutaneous leishmaniasis originating from this region (Silveira, personal communication).

The present study represents the first systematic study to examine, mainly through molecular methods, the repertoire of *Leishmania* species occurring in this region, and to our knowledge, revealing for the first time that *L.* (*V*.) *lindenbergi* (43.7%) and *L.* (*V.*) *lainsoni* (34.4%) are the main ACL agents in the BMR, followed by *L.* (*L*.) *amazonensis* (12.5%) and *L*. (*V.*) *braziliensis* (9.4%). From an eco-epidemiological point of view, it is interesting to note that these *Leishmania* spp. enzootics in the BMR remain established in residual forest fragments with ecological conditions, such as the presence of phlebotomine potential vectors^37^ ^,^ ^38^, which favor *Leishmania* life cycles. Clinical and socioeconomic data show ACL in the BMR as a predominantly accidental disease with an occupational/eco-touristic character, since it has mainly been associated with middle-aged men exposed to peri-urban forest environments. Occasional ACL cases of patients with no history of forest exposure (such as elderly housewives) have drawn attention to its potential for intra/peridomiciliary transmission.

The adaptation of *L.* (*V.*) *lindenbergi* and *L.* (*V.*) *lainsoni* enzootic cycles to the current ACL ecological scenario in the BMR is interesting. These species are together responsible for the majority (81.3%) of all ACL cases examined in this study. While *L.* (*V.*) *lainsoni* has already been found in the Brazilian Amazon States of Pará, Amapá^39^, Rondônia^40^, and Acre^41^ as well as in other South American countries/territories, such as Peru^42^, Bolivia^43^, Colombia^44^, Suriname^45^, French Guiana^19^, and Ecuador^46^, *L.* (*V.*) *lindenbergi* has, until recently, not been recorded outside its type-locality, the BMR. The first report of *L.* (*V.*) *lindenbergi* causing ACL occurred in Rondônia State, Brazilian western Amazon region^36^. Underreporting of ACL due to this parasite is possible, since some methodologies currently employed for *Leishmania* identification may not distinguish this species from others. Therefore, increasing efforts to develop novel techniques for species identification in other Amazonian regions may expand our knowledge on the geographical range of *L.* (*V.*) *lindenbergi*. 

In this study, we record the first three cases of ACL due to *L.* (*V.*) *braziliensis* in the BMR, with strains from the municipalities of Belém, Benevides, and Castanhal being compatible with the MHOM/BR/1975/M2903 RNAPOIILS-PCR-RFLP profile, thus, distinct from that of *L.* (*V.*) *lindenbergi*. The ecological scenario of *L.* (*V.*) *braziliensis* is not yet well understood in this region, as the well-known vectors *Psychodopygus wellcomei/Psychodopygus complexus* have not yet been recorded in surveyed forest fragments in the BMR^11^ ^,^ ^37^ ^,^ ^38^. Alternative transmission (most likely by other ‘psychodopygians’), thus, cannot be ruled out. *Psychodopygus davisi*, for instance, is a very active human-biting phlebotomine species present in the BMR that was found to be infected with *L.* (*V.*) *braziliensis* in the southern Pará State^47^.

In summary, the results of this study provide a better understanding of the ACL epidemiological scenario in the BMR. Strong ecological transformations have occurred in this region over the years, although these changes do not appear to limit the enzootic cycles of the *Leishmania* species already identified here.

## ETHICAL STANDARDS

Procedures involving humans were submitted and approved by the Comitê de Ética em Pesquisa - CEP (Ethics in Research Committee), under protocol CAAE 95080418.0000.0019. Procedures involving access to stored material of non-human vertebrates were submitted and approved by the Comissão de Ética no Uso de Animais - CEUA (Ethics in Animal Use Commission), under protocol CEUA/IEC/SVS/MS n.42/2018.
